# Evaluation of Genotoxic and Mutagenic Activity of Organic Extracts from Drinking Water Sources

**DOI:** 10.1371/journal.pone.0170454

**Published:** 2017-01-26

**Authors:** Ying Guan, Xiaodong Wang, Minghung Wong, Guoping Sun, Taicheng An, Jun Guo, Guoxia Zhang

**Affiliations:** 1 Guangdong Provincial Key Laboratory of Tropical Disease Research, School of Public Health and Tropical Medicine, Southern Medical University, Guangzhou, China; 2 Jiangxi Academy of Forestry, Nanchang, China; 3 Croucher Institute for Environmental Sciences, and Department of Biology, Hong Kong Baptist University, Hong Kong China; 4 Guangdong Institute of Microbiology, Guangdong Open Laboratory of Applied Microbiology, and Guangdong Provincial Key Laboratory of Microbial Culture Collection and Application, Guangzhou, China; 5 State Key Laboratory of Organic Geochemist, Protection, Guangzhou Institute of Geochemistry, Chinese Academy of Science, Guangzhou, China; CAS, CHINA

## Abstract

An increasing number of industrial, agricultural and commercial chemicals in the aquatic environment lead to various deleterious effects on organisms, which is becoming a serious global health concern. In this study, the Ames test and SOS/*umu* test were conducted to investigate the potential genotoxicity and mutagenicity caused by organic extracts from drinking water sources. Organic content of source water was extracted with XAD-2 resin column and organic solvents. Four doses of the extract equivalent to 0.25, 0.5, 1 and 2L of source water were tested for toxicity. All the water samples were collected from six different locations in Guangdong province. The results of the Ames test and SOS/*umu* test showed that all the organic extracts from the water samples could induce different levels of DNA damage and mutagenic potentials at the dose of 2 L in the absence of S9 mix, which demonstrated the existence of genotoxicity and mutagenicity. Additionally, we found that *Salmonella typhimurium* strain TA98 was more sensitive for the mutagen. Correlation analysis between genotoxicity, Organochlorine Pesticides (OCPs) and Polycyclic Aromatic Hydrocarbons (PAHs) showed that most individual OCPs were frame shift toxicants in drinking water sources, and there was no correlation with total OCPs and PAHs.

## 1. Introduction

Freshwater sources such as rivers, lakes and pond water are used as the primary source of drinking water, irrigation for agricultural purpose and for many human activities around the world. These surface water sources receive large quantities of wastewater from industrial, agricultural, and domestic sources; eventually get polluted and consequently pose a serious threat to human health and indigenous aquatic life. Purification of these surface waters are widely practiced with conventional water treatment processes like coagulation-flocculation, sedimentation, filtration and disinfection. Some reports specified that conventional wastewater purification processes do not effectively remove many chemical contaminants, and treatment may actually increase the mutagenicity/genotoxicity of wastewaters [1–3]. Moreover, these treatment processes are increasingly experiencing operational difficulties due to the widespread pollution of water resources [4]. it is particularly true in China due to the rapid economic growth, swift urbanization and industrialization over the last two decades. The 2005 report on the state of the environment in China showed that water resources still suffered from serious organic pollution, and nearly 60% of the monitored sections of major rivers did not comply with the water quality standards for drinking water supply [5].

Genotoxic/mutagenic compounds, including carcinogens, whether known or unknown, become the complex pollutant mixtures that can lead to adverse health effects on indigenous biota and humans [6]. Organic pollutants such as Organochlorine Pesticides (OCPs), Polycyclic Aromatic Hydrocarbons (PAHs) and Pharmaceuticals and Personal Care Products (PPCPs) are usually neglected because of their low concentrations during water quality monitoring, but these pollutants possess high persistence and bioaccumulative potency [7]. In addition, most of the discharged organic pollutants such as Persistent Organic Pollutants (POPs) are poisonous, which can become an acute or chronic hazard to human health. Some chemicals, such as PAHs and Polychlorinated Biphenyls (PCBs), are genotoxic agents in water, which might cause gene mutation and result in a series of harmful consequences, including malignancy and malformation [8].

Mutagenicity evaluation of sources of drinking water indicates a potential hazard caused due to contamination with putative toxicants. Assessment of mutagenicity/genotoxicity of surface water was carried out with different approaches. Mutagenicity/genotoxicity assays are classified into two major categories: bacterial assays and aquatic organism/plant assays [9]. Widely used bacterial assays include the Salmonella mutagenicity test, the SOS Chromotest and Salmonella umu-test; and aquatic organism/ plant assays include the micronucleus assay, 32P-post- labeling, the comet assay and the alkaline unwinding assay. Since 1980, a variety of bioassays and analytical methods have been used to assess genotoxicity of surface water. Among all bioassays, Salmonella mutagenicity assay in particular has been widely used to detect mutagenic activity in complex environmental mixtures such as surface waters, especially river waters. The *Salmonella* mutagenicity assay, namely Ames and SOS/*umu*-test are both short-term bacterial tests based on the detection of chemically induced DNA lesions that could lead to DNA mutations (the Ames test) or SOS response to bacterial strains that have been genetically developed. These assays help in evaluating the specific relation of DNA lesions with mutagenesis and genotoxicity [10]. The Ames test, in particular, has been widely used to detect mutagenic activity in complex environmental mixtures such as surface waters, especially river waters [9]. This test is based on the detection of histidine-independent revertants in selected *Salmonella* strains after exposure to certain mutagens. The strains TA98 and TA100 have been reported to be very sensitive, responding to a broad range of mutagenic compounds, and are suggested as the basic strains according to DIN38415-4 (1999) and ISO 16240 standards (2002). The SOS/*umu* test system is a genotoxicity test with the bacteria *Salmonella typhimurium* TA1535/pSK1002, which carries the plasmid pSK1002 with the *umu*-C-*lac*Z fusion gene. The *umu*-C operon is induced in response to genotoxic lesions in the bacterial DNA. Since the *umu*-C operon is fused with the *lac*Z-gene for β-galactosidase, the induction of the *umu*-C operon can be easily assessed by the determination of β-galactosidase activity related to bacterial density. The comparison of the induction ratio (IR) of the *umu*-C gene on exposure to water samples to its spontaneous activation gives a measure of genotoxicity. The SOS/*umu*-test has been used for the determination of the genotoxic potential of drinking water contaminated with wastewater. For this purpose the test has been standardized according to DIN and ISO (DIN 38415 1999; ISO 13829 2000) [11].

These two tests are complementary [12], and have been used together in order to broaden the detection capacity and to evaluate the overall genotoxicity in the present study. Other studies focus on concentrated samples and metabolic activation [13], or with un-concentrated samples with or without metabolic activation [14, 15]. White and Rasmussen (1998) [16] indicated that putative genotoxins in surface waters is primarily direct-acting; i.e., S9 addition does not enhance the response. Considering this, in our study, the genotoxicity of all samples was investigated by combining the Ames with SOS/*umu*-test without S9 mix.

The Pearl River Delta (PRD) is the most developed and densely populated area in Southern China [17]. Due to serious environmental pollution from industry and agriculture, the drinking water source is highly contaminated with organic wastes causing a threat to millions of humans living around this area [6]. In our study, we sampled six locations, which were chief drinking water source for the residents around this area. The locations included the Jianggao (JG), Yagang (YG), Xicun (XC), Yajisha (YJS) sections of Liuxi river, the Liuwuzhou (LWZ) section of Dongjiang river and the Shunde (SHD) section of Beijiang river. In order to protect of the aquatic ecosystem and safety of drinking water, a large-scale campaign has been launched by the central government, together with local authorities. Presently, many physical and chemical parameters have been under routine monitoring; however, these parameters could not demonstrate the adverse effects of chemicals in mixtures. Several genotoxicity studies have been carried out in China [18–20] (Nanjing, Chongqing, the Yangtze Estuary, Taihu and Shanghai) and Korea, Argentina, India and Brazil [21–24]. To our knowledge, no study has been focused on evaluating the potential toxicity of organic extracts of drinking water source in the Pearl River area.

Organic pollutants in rivers included Dichlorodiphenyltrichloroethane [25, 26], OCPs [27, 28], PCBs, Hexachlorocyclohexanes (HCHs) and PAHs [29, 30]. These organic pollutants are classical examples of POPs, with worldwide concern owing to their persistence, bioaccumulative ability, and potential negative impacts on humans and animals [31]. We have previously reported the toxicological significance of organic pollution of the drinking water source and its safety assessment to human health [32, 33].

In this study, genotoxic potentials of organic extracts in the drinking water sources from wet (from April to July, 2008) and dry seasons (from December 2008 to February 2009) were tested. DNA damage and mutation caused by organic extracts in the water samples from different sampling sites were investigated with the Ames test and SOS/*umu* test. The objectives of our study includes: (1) examination of the mutagenicity and genotoxicity of the organic extracts of drinking source water; and (2) evaluation of the relative contributions of OCPs and /or PAHs to the total mutagenicity and genotoxicity detected in the drinking source water. Our research findings on the genotoxic potential of drinking water sources in the study area thus provide all the in-depth basic information necessary for further risk assessment of the source water and enlighten the concerned authorities regarding necessary action.

## 2. Materials and Methods

### 2.1 Ethical statement

This study was approved by the Ethical Committee of Southern Medical University, Guangzhou. All sample collection sites provided by authority organization in local government (Water Resources Department of Guangdong Province). The field studies did not involve endangered or protected species.

### 2.2 Chemicals and media

The chemicals Dimethylsulfoxide (DMSO), 4-nitroquinoline-*N*-oxide (4-NQO), 2-mercaptoetanol, amberlite XAD-2 resins and o-nitrophenyl-β-D-galactopyranoside (ONPG) were purchased from Sigma (St. Louis, USA). All other chemical reagents were of the highest commercial quality available. A further cleaning was conducted for XAD-2 resins with methanol and dichloromethane in a Soxhlet extractor system. Then, the pre-cleaned XAD-2 resins were soaked in methanol. The resins column was eluted with 500 mL deionized water before use. Other solvents and chemicals were of analytical grade.

### 2.3 Study sites and water sample collection

Water samples were collected from the source water at 6 different sampling sites including JG, YG, XC, YJS, LWZ and SHD in the wet period (from April to July, 2008) and the dry period (from December 2008 to February 2009). The geographical locations of water sample collection area are marked in the supplementary file (Table C in S1 File). At each sampling site, six water samples (60 L per sample) were collected in the wet and dry periods for later analysis. Water samples were brought to the laboratory and stored for 24 h at 4°C. Then each water sample was passed through a glass column with non-polar neutral resin (XAD-2) to adsorb organic pollutants. The XAD-2 was pre-cleaned by consecutive Soxhlet extractions with acetone, n-hexane and methanol (10 h each) and kept in methanol until field application in the batch-wise extraction. The velocity of flow was controlled at 30–40 mL/ min. Organic matter was eluted with 300 mL dichloromethane /n-hexanex (V:V, 85:15) and 200 mL acetone (rate of 3–5 mL/ min). The organic solvents were evaporated to a small volume at 40°C under reduced pressure with a rotary evaporator and then dried by blowing with a nitrogen stream. The dry residue was re-dissolved in 3.0 mL DMSO. Further dilutions were performed as necessary. Samples were stored in a freezer at -20°C until use.

### 2.4 *Salmonella typhimurium* mutagenicity assay: Ames test and SOS/*umu*-test

The test strains, *Salmonella typhimurium* histidine auxotrophs TA98 and TA100 were obtained from Wuhan Institute of Environmental Medicine, Tongji Medical College and Huazhong University of Science and Technology. The procedure of the Ames test without metabolic activation was carried out as described by Legault et al. [34]. The overnight grown cell suspensions (approximately 10^8^ cell/ mL) of TA98 and TA100 were prepared. Four dilutions (0.25, 0.5, 1.0 and 2.0 L equivalent source water) of water samples were used for toxic analyses. The diluted test sample (0.1 mL) and bacterial culture (0.1 mL) were thoroughly mixed with 2.5 mL of molten top agar (0.6% agar and 0.5% NaCl) and poured over the surface of Vogel–Bonner minimal agar plate (1.5% agar, 0.4% glucose, 2% K_2_HPO_4_, 0.7% NaNH_4_HPO_4_·4H_2_O and 0.04% MgSO_4_·7H_2_O). The top agar layer was allowed to solidify, and the plates were incubated in the dark at 37°C for 48 h. Subsequently, the number of revertants and surviving colonies formed per plate, against a background lawn of growth, were counted. 4-Nitroquinoline 1-oxide was used as positive controls and sterile distilled water as a negative control. The mutagenic effect was evaluated from the number of revertant colonies per plate. The plates were prepared in triplicate for every test sample, and the result presented was the mean of triplicate observation (± standard deviation). The sample was considered as a positive response only if the mutation rate (MR) ≥2.00 (MR = mutant colonies on test plate/spontaneous mutant colonies on negative control plate).

The SOS/*umu*-test was performed in test tubes without S9 metabolic activation according to ISO standard with modifications (ISO 13829: 2000). The test strain, *S*. *typhimurium* TA1535/pSK 1002 was obtained from Deutsche Sammlung von Mikroorganismen und Zellkulturen, Braunschweig, Germany. The strain was grown in TGA medium (containing 20μg ampicillin per mL). The overnight culture was diluted to OD _595_ = 0.28 with fresh TGA medium and incubated at 37°C for 3 h. The incubation mixtures consisted of the source water concentrations (equal to 0.25, 0.5, 1 and 2 L of original water) dissolved in DMSO 100μL, bacterial culture 600μL. 4-NQO (0.5 μg·mL^-1^) was used as a positive control, and DMSO as a negative control. After 3 h exposure, 2.7 mL B-Buffer (pH 7.0) with 10 μL chloroform were added to the mixture and incubated with shaking at 150 rpm for 10 min, then 500 μL ONPG solutions (dissolved in phosphate buffer) was added. The genotoxic activities were expressed in β-galactosidase units and in enzyme induction ratios (IR) related to control samples treated with DMSO. Water samples were classified as non-genotoxic if the IR<1.5, as marginally genotoxic if the IR ranges between 1.5 and 2.0, as genotoxic if IR>2, and dose-response relation was observed. All concentrations were carried out in triplicate.

### 2.5 Statistical analyses

Statistical analyses of data were performed by using One-way analysis of variance (ANOVA), and Pearson correlation and linear regression were performed by IBM SPSS 19.0 software. ANOVA was used to compare the mutagenic potency or induction ratio in two seasons, and genotoxicity induced by all the control and water samples. Pearson correlation (coefficient) and linear regression analyses were used to investigate the relationship between mutagenic potency or IR and OCPs or PAHs of source water.

## 3. Results and Discussion

### 3.1 Mutagenic potency of organic extracts of drinking water source

**Fig 1:**
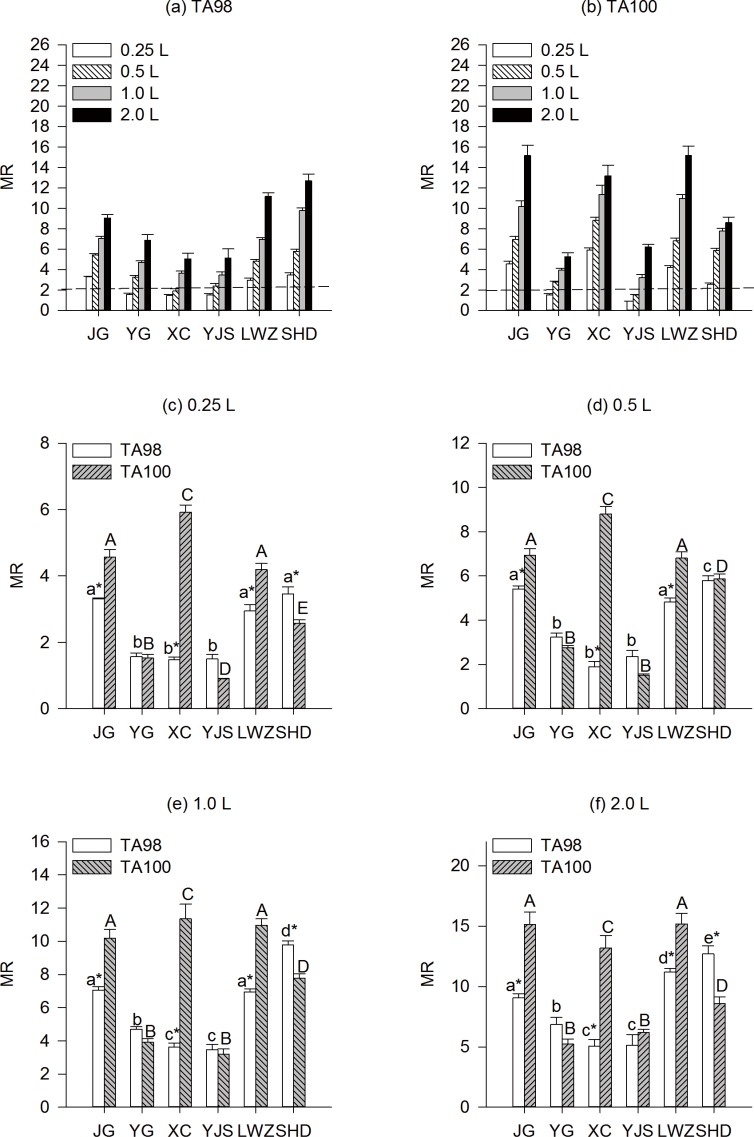
**Mutagenicity of TA98 (a) and TA100 (b) detected by Ames test without S9 mix in organic extracts from source water at dose 0.25L (c), 0.5L (d), 1.0L (e) and 2.0L (f) collected during the wet season at six sampling locations in Guangzhou drinking water source**. Lower-case letters and upper-case letters indicated pair-wise comparison in TA98 and TA100 from different sampling regions at the same water level. *indicated the significant difference compared with the strain TA100 at the same water level from the same sampling region.

**Fig 2:**
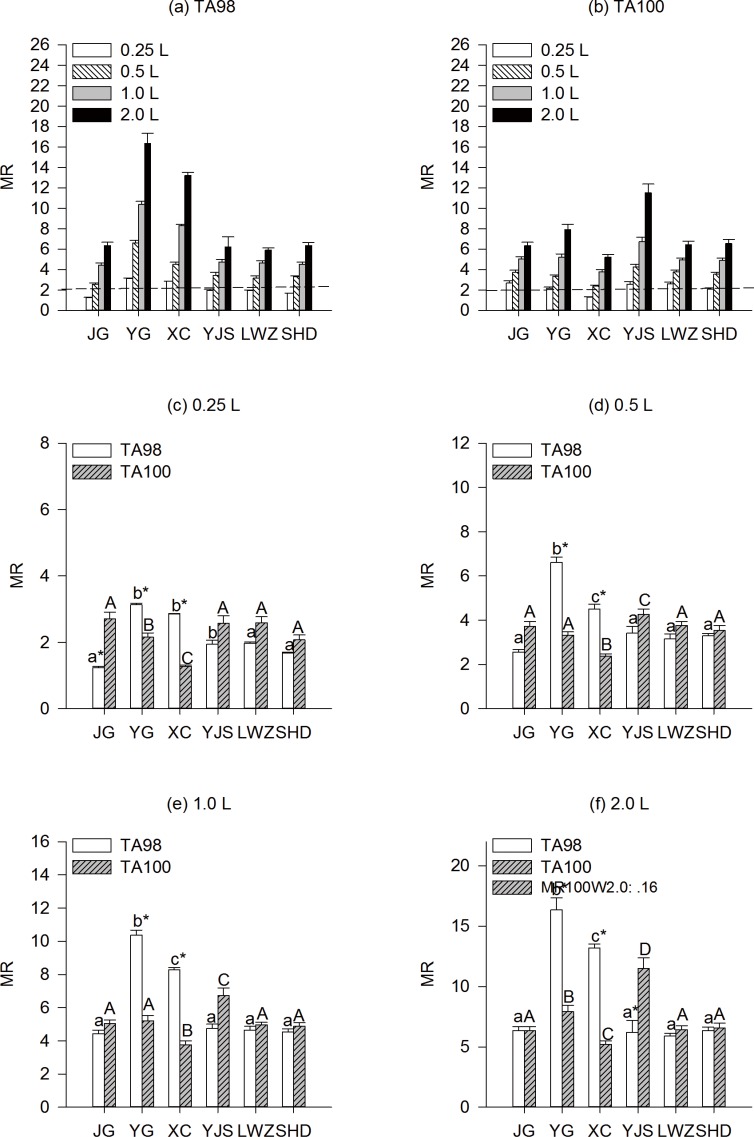
**Mutagenicity of TA98 (a) and TA100 (b) detected by Ames test without S9 mix in organic extracts from source water at dose 0.25L (c), 0.5L (d), 1.0L (e) and 2.0L (f) collected during the dry season at six sampling locations in Guangzhou drinking water source**. Lower-case letters and upper-case letters indicated pairwise comparison in the TA98 and TA100 in the different sampling regions at the same water level, respectively. *indicated the significant difference compared with the strain TA100 at the same water level in the same sampling regions.

The mutagenicity of organic extracts without metabolic activation (absence of S9 fraction) was investigated in *S*. *typhimurium* test strains TA98 and TA100. The Ames test results of water samples on the wet and dry seasons were summarized in (Fig 1) and (Fig 2). All samples produced frameshift mutations (in TA98 strains) and base pair substitution mutagenic potentials (in TA100 strains) with different levels. Additionally, we observed an increasing tendency of mutagenic potency with increasing water dose samples from all six locations. In strain TA98 without S9 mix, water samples collected during the wet season from JG, LWZ and SHD displayed mutagenicity with the least dose of 0.25 L source water per plate, while samples from YG and YJS showed mutagenicity at 0.5 L source water per plate, and sample XC displayed mutagenicity at 1.0 L source water per plate (Fig 1A). In strain TA100 without S9 mix, only samples YG and YJS showed negative as the dose was 0.25 L source water per plate. Other samples showed positives as the dose was 0.25 L source water per plate (Fig 1B). Sample SHD showed highest mutagenic potentials at all water levels in strain TA98 (Fig 1C–1F), and sample YJS displayed the lowest mutagenic potentials at all water levels in strain TA100 (Fig 1C–1F). Compared to TA100, strain TA98 was less sensitive for detecting potential mutagens at sample JG, XC and LWZ at all water levels; however, TA98 was more sensitive for detecting potential mutagens at sample SHD at high water level (>1L source water per plate).

In strain TA98 without S9 mix water samples collected during of dry season, samples from YG and XC demonstrated frameshift mutagenic potentials at the lowest dose of 0.25 L source water only; as other samples showed mutagenicity when the dose was up to 0.5 L source water per plate (Fig 2A). In strain TA100 without S9 mix, only sample XC had no frameshift mutagenic potential when the dose was 0.25 L per plate (Fig 2B). Sample YG and XC displayed high mutagenic potentials at all water levels in strain TA98 (Fig 2C–2F); however, sample XC displayed the lowest mutagenic potentials at all water levels in strain TA100 (Fig 2C–2F). Compared to TA100, strain TA98 was more sensitive for detecting potential mutagens at sample YG and XC at all water levels. Most mutagens among the organic extracts of drinking source water were capable of inducing frameshift mutations, and our results are consistent with other previously reported studies [18, 35–37]. More frameshift mutations had been found in source water and tap water from other different locations in China[38–39].

### 3.2 Genotoxicity of organic extracts of drinking water source

**Fig 3:**
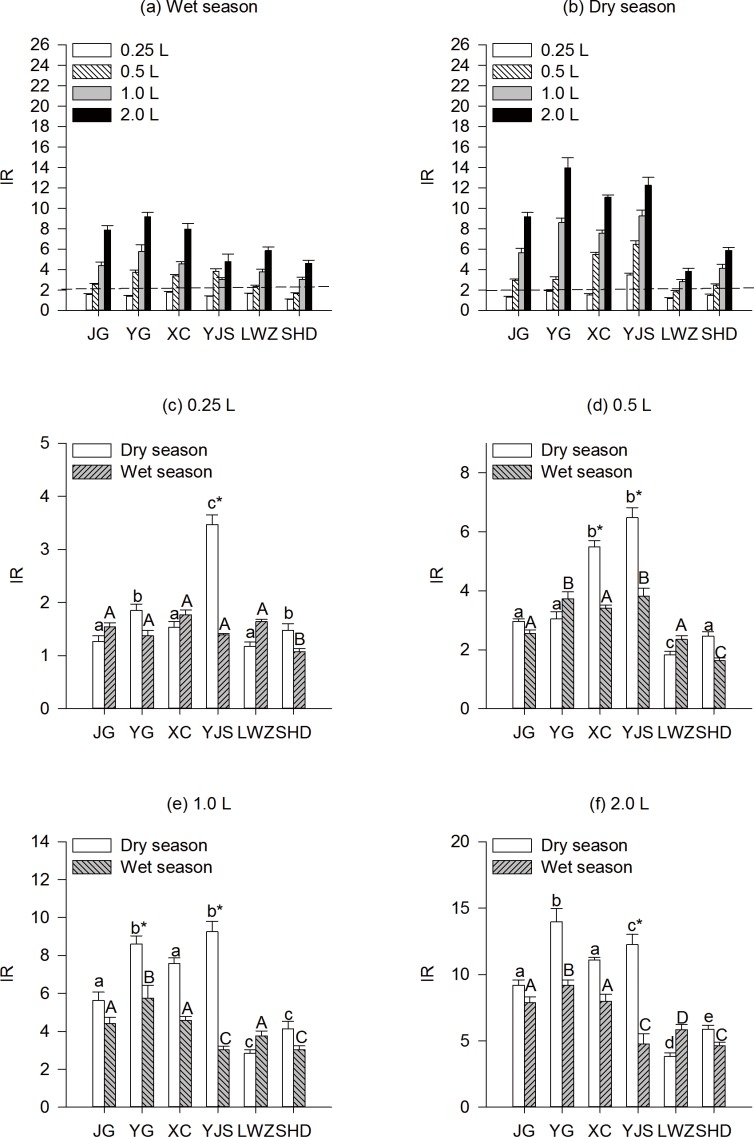
Genotoxic activity of organic extracts in water samples detected by SOS/*umu* test from six sampling locations in Guangzhou drinking water source. Lower-case letters indicated pair-wise comparison in the dry season in different sampling regions at the same water level and upper-case letters indicated pair-wise comparison in the wet season in different sampling regions at the same water level. *indicated the significant difference from the wet season.

The DNA damage effect of organic extracts was tested with *S*. *typhimurium* TA1535/pSK1002 in the absence of metabolic activation (Fig 3). Taking the results sample by sample, in fact, no sample was able to induce genotoxicity under 0.25 L water in wet season samples (Fig 3A), and only sample YJS could induce genotoxicity at dose 0.25 L source water per tube on dry season samples (Fig 3B). Wet season sample SHD and dry season sample LWZ were non-genotoxic with doses as low as 0.5 L water, and other samples showed genotoxicity on wet and/or dry seasons when the dose was 0.5 L per tube (Fig 3A and 3B). Compared with the wet season, the genotoxic activity was higher in the dry season from water samples collected from region YJS at all water levels (Fig 3C–3F). Because no industrial activity in this riverside according to our investigation. It would be reasonable to postulate that human activity plays an important role in the deterioration of water pollution in wet season. Therefore, organic extracts from all water samples collected from various regions in our study produced DNA damages according to the results of SOS/*umu*-test (Fig 3). Accordingly, much research analysis of worldwide drinking water sources have reported potential genotoxic activity [15, 19, 20]. It is difficult to compare these results because they are highly dependent on many other external parameters, such as the composition of the sampling water, the surroundings, time of sampling and the source of wastewater contamination.

### 3.3 Comparison of toxicity potency between Ames and SOS/*umu* tests during dry season

**Fig 4:**
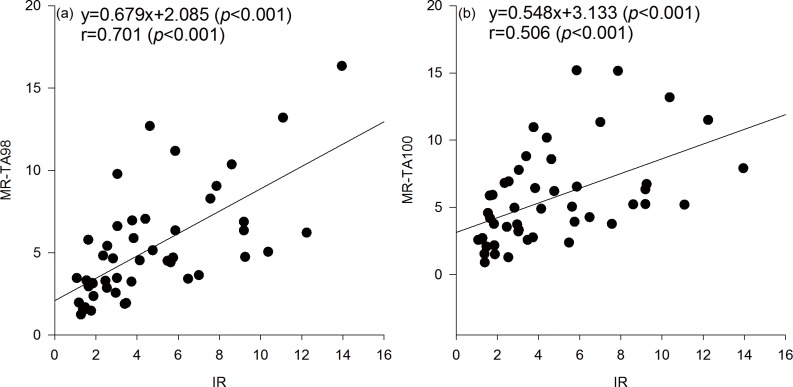
Relationship between the mutagenic ratio on TA98 without S9 activation and IR by SOS/*umu* test without S9 activation.

Upon comparing the toxicity of dry season water samples collected from different locations the sample from YG showed the highest mutagenic potency on TA98 (Fig 2A), and it was consistent with the findings observed in SOS/*umu*-test that YG water sample had the greatest IR. YG is near from major industrial locations, animal farming district and residential areas, which help us infer that these activities discharged mostly untreated sewage and pollutants into YG river. Regardless of water doses and locations, a significant correlation (r = 0.701, *p*<0.001 and r = 0.506, *p*<0.001) by Pearson correlation analysis was obtained between IR and MR from the strains TA98 and TA100 in drinking water sources, respectively (Fig 4). Moreover, a linear relationship was observed between IR and MR obtained from strains, TA98 (regression coefficient = 0.679, *p*<0.001) and TA100 (regression coefficient = 0.548, *p*<0.001). One possible explanation for this observation is that most compounds that introduce base pair frameshift mutations in the strain TA98 also caused DNA lesions in the SOS/*umu*-test. The results obtained in this study displayed a similar trend as those derived from the Ames and SOS/*umu*-test. Our findings are consistent with two previous studies [36, 39], but different from four other reported studies [35, 40–42]. Among all these previous studies, a great variation of source water sampling was noticed, which could be the possible reason for variation in the results. Hospital wastewater was used in the study conducted by Jolibois et al. [41], whereas chemical compounds were used by Leite et al. [35]. The studies of Fang et al. [42] and Penders and Hoogenboezem [43] also used surface water, but the difference was probably due to different chemical compositions of surface water samples.

### 3.4 Relationship between genotoxicity and OCPs or PAHs

The concentration of OCPs and PAHs from the water sources were collected in wet and dry seasons were analyzed (S1 Fig). In this study, regardless of water dose and seasons, several individual OCPs caused frameshift mutagenicity, in which a negative correlation existed between MR detected with the strain TA98 and δ-HCH (r = -0.839, *p* = 0.037), γ-HCH (r = -0.903, *p* = 0.014), Methoxychlor (r = -0.908, *p* = 0.012). Only α-HCH showed substitution mutation. The result was different from the previous study in that only endosulfan showed significant positive correlation (*p* = 0.004) with the mutagenic ratios detected with the strain TA98 [36]. Methoxychlor appears to show inhibiting action to substitute mutagenic potency and genotoxicity, which is consistent with previous studies conducted with Ames assay mutagenicity tests [9, 44, 45] without metabolic activation. For individual OCPs, Heptachlor showed significant positive correlation with the mutagenic ratios detected with strain TA98 (r = 0.859, *p* = 0.032), which was inconsistent with the results obtained from the International Agency for Research on Cancer that heptachlor was mutagenic in plants but not in bacteria. However, negative correlation was also detected between Heptachlor and IR (r = -0.894, *p* = 0.016), which may be attributed to heptachlor that does not cause DNA breakage (IARC, 1987). On the contrary with Heptachlor, γ-HCH showed negative in frameshift mutagenic potency (r = -0.903, *p* = 0.014) in the strain TA98, and positive in genotoxicity (IR) (r = 0.833, *p* = 0.040). It is likely that γ-HCH inhibit components of the SOS-system or the β-galactosidase. This is supported by the fact that xenobiotic contaminations are high in those samples, which is mutagenic, but not or weakly in SOS inducing samples [46]. The complexity of the mixtures can also elevate SOS-inhibiting compounds. Moreover, MR detected from TA98 showed positive correlations with other individual OCPs and PAHs as follows: TSS (r = 0.846, *p* = 0.034), DDD (r = 0.949, *p* = 0.004), DDTs (r = 0.874, *p* = 0.023), DDE (r = 0.911, *p* = 0.012), Endrin-aldehydes (r = 0.898, *p* = 0.015), γ-chlordane (r = 0.889, *p* = 0.028), Chlordane (r = 0.882, *p* = 0.020), Acenaphthene (r = 0.924, *p* = 0.008). Negative correlations existed between IR and Methoxychlor (r = -0.879, *p* = 0.021), Benzo(k)fluoranthene (r = -0.822, *p* = 0.0.45), or Benzo(a)pyrene (r = -0.864, *p* = 0.026). There was a positive correlation between α-HCH and MR detected from TA100 (r = 0.948, *p* = 0.004), but no significant correlations were observed between PAHs and mutagenic potency of TA100 in the present study, although PAHs can induce both mutagenic and genotoxic potency [46, 47]. This may be because PAHs are indirect-acting mutagen, which need metabolic activation in order to exert its effect [48, 49]. Moreover, the interaction between different organic matters in a complex mixture plays a critical role in induction of mutagenicity.

A recent preliminary report on chemical analysis and biological assays on irrigative wastewater (Shijiazhuang city) indicates the presence of genotoxic chemicals that can harm humans due to its accumulation in the food chain [50]. Moreover, some retrospective studies revealed a significantly higher rate of cancer related mortality in wastewater irrigation area compared with control area. Collectively, our findings on the genotoxic potential of drinking water sources alarms towards improvement of wastewater management and implementing efficient purification systems to improve the human health.

## 4. Conclusions

Ames and SOS/*umu* tests indicated that organic extracts of drinking source water collected from the Guangzhou area induced mutagenicity and genotoxicity. The linear regression analyses indicated that TA98 mutagenic potency was significantly correlated with genotoxicity expressed as IR. Although many individual OCPs might contribute to the mutagenicity observed with the test strain TA98 without S9 mix, no significant correlations were found between mutagenicity and concentrations of total OCPs. OCPs might not be the dominant contaminants for the mutagenicity detected in drinking water sources.

In general, our study showed that all the tested source waters could induce genotoxic effects. However, it is difficult to determine the exact chemical responsible for genotoxic effects from the complex mixture displaying the predominant mutagenic and genotoxic activity. More studies need to be carried out with a careful analytical approach to unravel the exact identity and quantity of the compounds from the toxic complex mixture responsible for genotoxicity. This difficult task will be necessary to identify the sources of toxic contaminants and thus be able to take preventive and/or curative measures in order to limit the toxicity in the drinking water sources.

## Supporting Information

S1 File 3 tables: Tables A and B are raw data of genotoxicity and mutagenicity. Table C is geographical coordinates for all of the six areas where the field studies were performed.
(DOCX)

S1 Fig The concentration of OCPs and PAHs in the drinking water from six sampling site in Guangzhou. (EPS)
