# Supplementary material for: Evaluation of Genotoxic and Mutagenic Activity of Organic Extracts from Drinking Water Sources
Source: PLoS One. 2017 Jan 26;12(1):e0170454. doi: 10.1371/journal.pone.0170454 (PMC5268787; doi:10.1371/journal.pone.0170454)
Supplement: S1 File — 3 tables: Tables A and B are raw data of genotoxicity and mutagenicity. Table C is geographical coordinates for all of the six areas where the field studies were performed. (DOCX) [file pone.0170454.s001.docx]

**Supplementary data:**

**Table A Induction of revertants by drinking source water organic extracts from six sampling sites in Guangzhou in the Ames test without S9 mix**

| Sampling site | Doses  (L/Plate) | Dry season | | Wet season | |
| --- | --- | --- | --- | --- | --- |
|  |  | TA98 | TA100 | TA98 | TA100 |
| YG | 0.25 | 91±13 | 222±35 | 46±8 | 159±12 |
|  | 0.5 | 192±24 | 342±27 | 94±5 | 284±32 |
|  | 1.0 | 300±29 | 537±36 | 137±13 | 403±37 |
|  | 2.0 | 474±21 | 814±44 | 199±26 | 539±51 |
| JG | 0.25 | 56±9 | 138±12 | 146±18 | 513±85 |
|  | 0.5 | 116±12 | 256±12 | 238±36 | 763±64 |
|  | 1.0 | 288±14 | 406±13 | 310±24 | 983±20 |
|  | 2.0 | 372±6 | 560±21 | 398±52 | 1143±61 |
| XC | 0.25 | 107±5 | 257±35 | 56±7 | 77±8 |
|  | 0.5 | 168±24 | 427±40 | 72±6 | 133±11 |
|  | 1.0 | 309±20 | 673±40 | 192±49 | 277±25 |
|  | 2.0 | 493±24 | 1150±60 | 32.0±8.0 | 537±32 |
| YJS | 0.25 | 88±3 | 292±56 | 66±2 | 427±40 |
|  | 0.5 | 155±9 | 402±26 | 104±9 | 647±51 |
|  | 1.0 | 215±18 | 544±12 | 152±12 | 950±79 |
|  | 2.0 | 281±16 | 684±37 | 226±21 | 1413±185 |
| LWZ | 0.25 | 89±12 | 280±40 | 130±12 | 363±40 |
|  | 0.5 | 143±12 | 406±44 | 212±36 | 590±40 |
|  | 1.0 | 211±18 | 536±33 | 306±36 | 950±56 |
|  | 2.0 | 476±8 | 694±37 | 492±49 | 1317±89 |
| SHD | 0.25 | 149±19 | 224±33 | 152±23 | 278±24 |
|  | 0.5 | 254±21 | 382±25 | 254±21 | 634±15 |
|  | 1.0 | 205±13 | 528±42 | 430±42 | 840±28 |
|  | 2.0 | 288±17 | 706±45 | 558±65 | 927±42 |
| Positive control |  | 257±35 | 4636±405 |  |  |

Positive controls, 4-NQO (0.5μg/ml) for TA98 and TA100 without S9. All samples Significantly different from negative control group at *p*<0.05 (t-test).

**Table B Genotoxic activity in the SOS/*umu* test of organic extracts from different sampling sites in Guangzhou drinking water sources**

| Sites | Doses  (L/tube) | Dry season Wet season | |
| --- | --- | --- | --- |
|  |  | IU  (mean±sd) | IU  (mean±sd) |
| YG | 0.25 | 10.67±0.74 | 16.44±1.52 |
|  | 0.5 | 21.33±2.06 | 28.45±2.08 |
|  | 1.0 | 36.92±3.66 | 49.49±0.65 |
|  | 2.0 | 80.07±4.21 | 93.92±3.56 |
| JG | 0.25 | 7.15±0.81 | 23.85±0.18 |
|  | 0.5 | 16.6±0.42 | 39.40±0.21 |
|  | 1.0 | 31.59±0.29 | 68.13±0.27 |
|  | 2.0 | 51.57±0.05 | 121.55±0 |
| XC | 0.25 | 14.19±0.30 | 13.81±0.37 |
|  | 0.5 | 30.76±0.57 | 26.55±0.21 |
|  | 1.0 | 42.41±0.54 | 54.60±1.02 |
|  | 2.0 | 62.22±2.70 | 80.92±4.47 |
| YJS | 0.25 | 19.44±1.19 | 21.67±0.27 |
|  | 0.5 | 30.70±0.46 | 29.13±0.61 |
|  | 1.0 | 40.68±0.69 | 46.83±0.25 |
|  | 2.0 | 51.84±0.74 | 73.65±0.27 |
| LWZ | 0.25 | 6.55±0.56 | 25.4±0.79 |
|  | 0.5 | 9.13±0.31 | 36.42±0.21 |
|  | 1.0 | 14.19±0.34 | 58.17±0.81 |
|  | 2.0 | 21.83±0.56 | 90.52±0.27 |
| SHD | 0.25 | 11.06±0.37 | 21.98±1.53 |
|  | 0.5 | 18.65±0.70 | 28.61±3.41 |
|  | 1.0 | 25.19±0.60 | 33.71±1.27 |
|  | 2.0 | 32.91±2.66 | 77.58±2.74 |
| Positive control |  | 185.84±7.89 |  |

Positive controls, 4-NQO (0.5μg/ml) for TA98 and TA100 without S9. All samples Significantly different from negative control group at *p*<0.05 (t-test).

**Table C. Geographical coordinates (longitude/latitude) for all of the six areas where the field studies were performed.**

| Sample sites | **North latitude** | **East longitude** |
| --- | --- | --- |
| JG | **N23°17′34.98″** | **E113°13′45.62″** |
| YG | **N23°13′59.24″** | **E113°11′25.20″** |
| XC | **N23°08′39.86″** | **E113°14′14.04″** |
| YJS | **23°03′33.78″** | **E113°16′32.46″** |
| LWZ | **N23°07′40.04″** | **E113°40′56.41″** |
| **SHD** | **N22°48′24.94″** | **E113°17′20.59″** |
